# Age-related differences in the prevalence of premature ejaculation: taking a second and more detailed look

**DOI:** 10.1093/sexmed/qfae057

**Published:** 2024-09-02

**Authors:** David L Rowland, Zsuzsanna Kӧvi, Krisztina Hevesi

**Affiliations:** Department of Psychology, Valparaiso University, Valparaiso, IN 46383, United States; Institute of Psychology, Károli Gáspár University of the Reformed Church, Budapest 1091, Hungary; Institute of Psychology, Eötvös Loránd University, Budapest 1075, Hungary

**Keywords:** premature ejaculation, acquired premature ejaculation, lifelong premature ejaculation, age, prevalence, diagnostic criteria for PE

## Abstract

**Background:**

Research indicates an inconsistent relationship between age and the prevalence of premature ejaculation (PE), with studies reporting an increase, decrease, or no change with age.

**Aim:**

To reexamine the possible relationship between age and PE prevalence, implementing methodological improvements that enhance the likelihood of detecting real effects.

**Methods:**

From a sample of 2772 men, we analyzed a subset of 418 classified as having probable or definite PE based on the Premature Ejaculation Diagnostic Tool. We not only analyzed men with lifelong PE (LPE; n = 316) and acquired PE (APE; n = 102) separately but assessed prevalence differences across age groups using an omnibus measure to establish PE status and specific PE diagnostic criteria, individually and in multifactorial combination.

**Outcome:**

Prevalence of PE in younger vs older men.

**Results:**

LPE, but not APE, showed age-related differences in prevalence, with LPE being lower in the higher age group. This pattern was most discernible when a multifactorial approach was used to establish PE status.

**Clinical Translation:**

Older men may be less distressed about their dysfunction or may benefit from diminishing ejaculatory function with age.

**Strengths and Limitations:**

This cross-sectional study used an improved methodology to detect age-related differences in PE prevalence. Future studies would benefit from a larger sample size that enables a breakdown of prevalence using a greater number of age categories.

**Conclusion:**

According to an improved methodology, men with LPE showed a decline in prevalence with aging. A methodology aimed at exploring this relationship should—at the very least—not only distinguish between LPE and APE subtypes but also consider using a multifactorial method of determining PE status that includes a measure of bother/distress.

## Introduction

Premature ejaculation (PE) reportedly occurs in about 5% to 10% of the male population.^1^ Although terminology differs, all contemporary definitions of PE include 3 dimensions: (1) ejaculation upon minimal stimulation, commonly defined in terms of a short ejaculation latency (EL); (2) a lack of ability to delay or postpone ejaculation (ie, lack of ejaculatory control); and (3) negative consequences, such as distress, bother, and/or avoidance of intimacy (ie, typically “bother/distress”).^2-6^

Whether the prevalence of PE varies with age has long been an unresolved issue. Given that ejaculatory function and penile sensitivity are known to decrease with age,^7^^,^^8^ early theorizing assumed that men with a history of PE might benefit from longer ELs as they age.^9^ Yet, several early empirical studies^10^^,^^11^ reported little or no variation in PE prevalence across age groups. Although the definitional criteria for PE were ill-defined when these early studies were conducted, a spate of pharmaceutically supported reviews on the topic reported these preliminary findings as “fact,” establishing the narrative that PE prevalence was not age related.^12-18^ Despite occasional early exceptions to the “rule,”^19^ this same conclusion was echoed years later in the 2015 International Consultation on Sexual Medicine report, which matter-of-factly stated that PE prevalence was not age related.^2^

However, such a conclusion ignores the numerous inconsistencies in studies examining this issue over the past 2 decades, with various studies reporting an increase, decrease, or no change in age-related prevalence.^20-27^ Given the current state of the literature, no conclusion regarding the relationship between age and prevalence can currently be justified with any degree of confidence.

Although few in-depth analyses on the issue of age and PE prevalence have been conducted within the past several years, 3 reports are worth noting. In a systematic review of the epidemiology of male sexual dysfunctions in Asia and Europe, Irfan et al^28^ reported that PE prevalence decreased with age in European but not Asian men. A recent probability study in Germany based on the latest *ICD-11* criteria showed that PE prevalence decreased with age, a pattern that was most pronounced when PE classification included a measure of bother/distress.^29^ Yet, a recent Danish probability study found no consistent relationship between age and PE prevalence.^30^ Thus, even these recent studies suggest that the age-prevalence issue remains unresolved.

### Age and prevalence: why the discrepancies?

So, how might such discrepant findings be explained? First, few of the previously cited studies were actually designed to explore the age-prevalence relationship as a significant goal within the context of an epidemiologic study; therefore, their methodologies were sometimes woefully inadequate. Second, prevalence studies have relied on different methodological strategies for classifying PE status—partly related to PE criteria that have varied over time and by definitions.

Yet, there are cogent hypothetical reasons supporting the idea that PE prevalence might change with age. For example, as postulated years ago,^9^ PE symptomology might *decrease* in men with lifelong PE (LPE) due to an age-related decrease in penile sensitivity and other physiologic changes resulting in increased ejaculatory latencies.^7^^,^^27^ Furthermore, given that men with acquired PE (APE) tend to develop PE symptomology later in life—and that such symptomology becomes increasingly common with age-related health issues—prevalence might actually *increase* with age in men with this PE subtype.^22^^,^^31-33^ In fact, PE prevalence patterns might be canceled out when men from both groups are combined within a single analysis.

While cross-sectional methodology—characteristic of nearly all the studies previously cited—is ill-suited for delineating life span changes in PE prevalence, longitudinal designs represent a near insurmountable challenge for studies of this type. Nevertheless, methodological improvements could readily be implemented in cross-sectional analyses that could boost confidence in the findings. For example, the prevalence of PE subtypes (LPE and APE) could be identified and analyzed separately; retrospective-prospective items could be included; and PE status could be defined in a variety of ways, such as single vs multiple criteria, as recently implemented in an analysis that revealed age effects.^29^

### Rationale and goals

Until a body of literature based on more controlled methodologies becomes available, the relationship between age and PE prevalence will remain unclear. In support of strengthening this literature, the current study reexamined the issue of age-related differences in PE prevalence, with the implementation of 2 important improvements.

First, we analyzed age-related prevalence in LPE and APE subtypes separately, an approach that acknowledges the varying etiologies for PE^6^ and eliminates the possibility of the 2 subtypes canceling out each other.

Second, we defined PE status by relying on multiple strategies, not only using an omnibus measure derived from the Premature Ejaculation Diagnostic Tool (PEDT),^34^ but also examining PE diagnostic criteria (ejaculatory control, EL, and bother/distress) separately and in combination with other criteria.^2-6^ In doing so, we could determine whether specific PE dimensions differ across age groups (eg, ejaculatory control or EL).

In accordance with the literature, we hypothesized (1) that men with LPE would show a lower prevalence in higher age groups and (2) that men with APE would show a higher prevalence in higher age groups.

## Method

### Participants

Participants were recruited by voluntary self-selection from July 2019 through February 2020 to complete an online survey on sexual health and behavior. The sample was recruited from the United States and other English-speaking countries (n = 699) and from Hungary (n = 3243), and it included men who responded to the research homepages, postings on several reddit.com forums, and unpaid social media and public announcements/advertisements. An additional anonymously coded group (not included in the data analysis) consisted of men attending a major Hungarian university (n = 134) who took an in-person version of the questionnaire for the sole purpose of establishing test-retest reliability of items after 4 to 6 weeks.

The completion rate was 81% of those who initially opened the survey. Among those completing the survey, men who had never had partnered sex were excluded, as were those who identified as asexual, transgender, or nonbinary or showed inconsistent responses as determined by embedded “attention checks.” The sample included 418 men with PE drawn from an overall sample of 2772 men, with a mean age of 37.7 years (SD, 12.9; range, 18-85). Of these 418 men, 316 reported LPE; 102 reported APE.

### Survey questionnaire

During survey development, in-person focus sessions were conducted in the United States and Hungary (n = 89; mean age, 23.8 years; range, 19-48). Participants reviewed questionnaire items for clarity and face validity and assessed the time required for survey completion. For Hungarian respondents, the questionnaire was translated to Hungarian and back-translated to English by professional translators. For standardized assessment scales embedded in the questionnaire, existing validated questions were used, with minor modifications as necessary (eg, replacing “intercourse” with “partnered sex”).

The first part of the online survey queried about demographic characteristics, as well as anxiety/depression for >6 months (continuously or intermittently) during the past 1 to 2 years (as a proxy for psychological health) and chronic medical conditions related to sexual functioning. The second part examined participants’ recent sexual and relationship histories and evaluated the frequencies of partnered sex, masturbation, and pornography use during masturbation. The third section addressed major sexual dysfunctions in men and, apropos to this study, included relevant items from the PEDT.^34^

### Measures

#### Determining PE classification

Multiple strategies were used to define PE status, a critical first step in determining prevalence. Specifically, we used a broad classification strategy using the PEDT and a classification based on specific PE symptomology, either used individually or in combination with other symptomology.^6^^,^^30^

*Broad PE classification* was based on a validated patient report outcome, the PEDT,^34^ with 3 of the 5 PEDT items focusing on ejaculatory control, the construct most central to PE.^35^^,^^36^ Response options ranged from 1 to 5, with higher scores indicating a higher probability of PE. Based on a scoring rubric with a proportional cutoff identical to the original instrument, scores of 13 to 15 represented “definite PE” and 11 and 12 represented “probable PE.” Internal reliability for the 3 items was 0.89.

*Individual PE symptomology* was based on current PE diagnostic criteria and included measures of ejaculatory control, EL, and bother/distress.^2-6^ Ejaculatory control was based on actual ejaculatory control PEDT scores (rather than *yes*/*no* categorizations of probable and definite PE). EL included estimated average and, separately, minimum latencies to ejaculation,^37^ with EL ≤3 minutes as a cutoff. Bother/distress was assessed with a single item rated on a 5-point scale (1 = never/almost never, 5 = almost always) asking how often the respondent felt bothered, distressed, frustrated, or guilty due to ejaculating quickly and before desired. This item was conceptually identical to the fourth question of the PEDT designed to assess bother/distress and was used only in combination with other PE symptomology, such as a lack of ejaculatory control.^29^

#### Determining PE subtype

After establishing “probable” or “definite” PE membership, respondents were further asked whether their condition had been present for all or nearly all of their sexual life (LPE) or whether it had developed more recently after a period of more typical or normal ejaculatory response/latency (APE).

#### Establishing age categories

Due to the limited sample sizes of men with LPE and APE, we could not distribute men across age decades of life (18-30, 30-40, 40-50, etc). Therefore, we used a combined theoretically and sample size–driven strategy to optimize detection of age-related differences in PE prevalence. For example, a rationale exists for an age-related decrease in older men with LPE as they experience diminished ejaculatory functioning.^3^^,^^7^ In addition, a rationale exists for an age-related increase in prevalence in men with APE, given that APE is secondary to other health problems that increase with age.^22^^,^^31-33^ Accordingly, we explored several 2- and 3-group age categorizations, ultimately selecting age grouping by <50 and ≥50 years because it met optimal age and cell size needs for adequate power (β − 1 = .80).

### Procedure

Ethics approval was obtained from the institutional review boards at our institutions in the United States and Hungary. The survey incorporated best practices in that no incentives or rewards were offered; completion time was <20 minutes; anonymity was guaranteed; safeguards prevented multiple submissions; and attention checks ensured internal reliability.^37^ Informed consent was obtained by participants’ checking boxes attesting to their current age ≥18 years and to their informed consent before accessing the questionnaire. Respondents could end participation at any time by closing the webpage.

### Data analysis

Because preliminary analyses indicated no or minimal differences between the US and Hungarian samples^38^ on relevant study variables, groups were combined for analysis. PE prevalence—as defined by a variety of univariate and multivariate methods—was examined in men with LPE and APE subtypes across age groups as reported previously, with the goal of elucidating possible age-related differences. We examined age-related differences in PE first using the *t*-test or *z*-test for proportions in just the definite PE group and then, to increase power, the definite + probable PE groups. Having established an a priori rationale for the direction of effects, we used 1-tailed tests (*P* ≤ .05) to maximize the detection of effects and minimize the type 2 error. Analyses were carried out with SPSS version 26.0 (IBM Corp).

## Results

### Description of the LPE and APE samples

Table 1 provides demographic information on men with LPE and APE, subdivided by categories of probable and definite PE. Most notable was the higher age of men with APE, although this difference was significant only for men in the probable PE group (*P* < .05).

**Table 1:** Comparison of men with PE subtypes on demographic variables.^a^

	**Probable PE**						**Definite PE**					
	**Lifelong**		**Acquired**		**Overall**		**Lifelong**		**Acquired**		**Overall**	
	**Mean**	**SD**	**Mean**	**SD**	**Mean**	**SD**	**Mean**	**SD**	**Mean**	**SD**	**Mean**	**SD**
Age, y	36.3_a_^b^	12.8	41.8_b_	13.5	37.5	13.1	38.2_a_	13.0	39.7_a_	11.4	38.6	12.6
Education	3.02_a_	1.47	2.59_a_	1.45	2.92	1.47	3.02_a_^b^	1.66	2.24_b_	1.67	2.82	1.69
Heterosexual, %	0.69_a_		0.72_a_		0.70		0.68_a_		0.58_a_		0.65	
Medical issue, %	0.20_a_		0.31_a_		0.22		0.22_a_		0.20_a_		0.21	
Anxiety, %	0.23_a_		0.27_a_		0.24		0.24_a_		0.27_a_		0.25	

^a^Values in the same row not sharing a subscript are significantly different at *P* < .05 according to a *t*-test or *z*-test for proportions. Tests are adjusted by Bonferroni correction.

^b^Indicates PE subtype group differences.

### Age-related differences in PE prevalence and classification strategy

PE classification strategies that relied on PEDT ejaculatory control classifications (*yes*/*no*: probable or definite), actual PEDT ejaculatory control score, or average/typical minimum EL (with PEDT *yes*/*no* classifications) demonstrated no clearly discernible age-related differences across age categories (see Table 2 for examples with ejaculatory control and ELs).

**Table 2:** Age-related prevalence in 2-group age categorizations.^a^

	**Definite PE**		**Probable and definite PE**	
	**Lifelong**	**Acquired**	**Lifelong**	**Acquired**
PEDT ejaculatory control				
≤50 y	13.49_a_ (1.27)	13.11_a_ (1.33)	11.15_a_ (2.06)	11.32_a_ (1.96)
>50 y	13.50_a_ (1.22)	13.89_a_ (1.36)	11.53_a_ (2.20)	11.17_a_ (2.39)
EL minimum, <3 min				
≤50 y	2.02_a_ (1.32)	2.20_a_ (1.49)	2.55_a_ (1.47)	2.54_a_ (1.55)
>50 y	1.81_a_ (1.21)	2.00_a_ (0.87)	2.33_a_ (1.29)	2.85_a_ (1.69)
PEDT + distress				
≤50 y	2.4_a_	0.8_a_	5.1_a_	1.6_a_
>50 y	0.4_b_	0.1_a_	0.6_b_	0.3_a_

Abbreviations: EL, ejaculation latency; PEDT, Premature Ejaculation Diagnostic Tool.

^a^ Data are presented as mean ± SD or percentage. Percentages were based on n = 2527 for the ≤50-year group and n = 474 for the >50-year group. Comparisons were made with a *t*-test or, for percentages, *z*-test for proportions. Shaded cells within columns that different subscripts indicate significant effects, *P* < .05.

A PE classification strategy that relied on multifactorial criteria that combined the PEDT classification with bother/distress demonstrated a predicted pattern for LPE: men with definite LPE (*z* = 2.78, *P* = .003) or probable + definite LPE (*z* = 4.36, *P* < .001) consistently showed a lower prevalence of PE in the higher age category. However, for men with APE, a consistent pattern of higher or lower prevalence in the higher age categories was not apparent (Table 2).

## Discussion

These findings may offer an explanation for, and perhaps a partial resolution to, the disparate findings across studies investigating the relationship between age and PE prevalence. Specifically, consistent with our first hypothesis, they suggest an age-related decrease in PE prevalence in men with LPE, being discernible primarily when multifactorial criteria were applied for establishing PE status. In contrast, an age-related pattern for APE was generally inconsistent. In this respect, our findings indicate the potential importance of analyzing LPE and APE subtypes separately, as combining subtypes might obscure specific subtype patterns/differences.

The results further indicate that how PE status is defined could affect the detection of age-related differences in PE prevalence. Specifically, using a simple univariate classification procedure for PE was less effective in detecting patterns than a multifactorial procedure that included “bother/distress.” A similar strategy of including bother/distress in the PE classification procedure yielded clear age-related differences in a German epidemiologic study,^29^ suggesting that a decrease in bother/distress may have been driving some of changes in age-related prevalence.^39^ In contrast with that study, however, our differences in prevalence across age groups were quite modest.

### Limitations

The gold standard for assessing age-related changes in PE prevalence necessitates a longitudinal design carried out over decades. Given the improbable nature of conducting such studies, unless supported as part of a national data repository on sexual health (eg, Denmark, Germany, Finland^27^^,^^29^^,^^30^), specific methodological strategies implemented within cross-sectional studies—as done in this study—may improve the detection of age-related differences in PE prevalence. Furthermore, although not included in our study, prospective-retrospective items that query men about their past and current symptomologies regarding PE might offer additional insight into age-related changes in PE symptomology. Finally, although our study included 418 men with PE, well-powered studies that track age cohorts by 10- to 15-year intervals (eg, 4-6 cohorts from ages 20 to 80 years) will likely require 2 or 3 times this number of respondents—a challenge for any study on a specific sexual dysfunction.

## Conclusion

The substantial disparity in the results of studies examining age-related differences in the prevalence of PE may be related to methodological shortcomings. Our findings suggest that exploration of the relationship between age and PE prevalence should—at the very least—distinguish between LPE and APE subtypes and consider using a multifactorial determination of PE status.^29^
